# Empowering self-reporting polymer blends with orthogonal optical properties responsive in a broader force range†

**DOI:** 10.1039/d0sc06140a

**Published:** 2020-12-08

**Authors:** Mengjiao Wu, Zhen Guo, Weiye He, Wei Yuan, Yulan Chen

**Affiliations:** Department of Chemistry, Key Laboratory of Mechanism Theory and Equipment Design of State Ministry of Education, Tianjin University Tianjin 300354 China yulan.chen@tju.edu.cn

## Abstract

Self-reporting polymers, which can indicate damage with perceptible optical signals in a tailored force range, are useful as stress-sensitive sensors. We demonstrate a simple approach to realize this function by embedding two distinct mechanophores — rhodamine (Rh) and bis(adamantyl)-1,2-dioxetane (Ad), in polyurethane/polylactic acid blends. The deformed blends generate red coloration and red chemiluminescence. Such a unique dual-responsive behavior was evaluated by solid-state UV-vis spectroscopy, macroscopic tensile tests with *in situ* RGB and light intensity analyses, which supported a stress-correlated occurrence of the ring-opening of Rh, the scission of Ad and the fluorescence resonance energy transfer process between the respective mechanochemical species. Complementarity stemming from the difference in properties and manifestations of the two mechanophores is essential. That is, the more labile Rh allows shifting the appreciable optical changes to a much lower force threshold; the transient nature and high dynamic range of mechanochemiluminescence from Ad map in real time where and when many of the covalently incorporated dioxetane bonds break; besides, the disrupted yet non-scissile structure of Rh acts as a fluorescent acceptor to effectively harvest chemiluminescence from ruptured Ad. The current strategy is thus empowering multi-functional mechano-responsive polymers with greatly improved sensitivity and resolution for multimodal stress reporting.

## Abstract 2

Probing bond scission process in a broader force range was realized by embedding mechanochromic rhodamine and mechanochemiluminescent 1,2-dioxetane in polymer blends.

## Graphical abstract

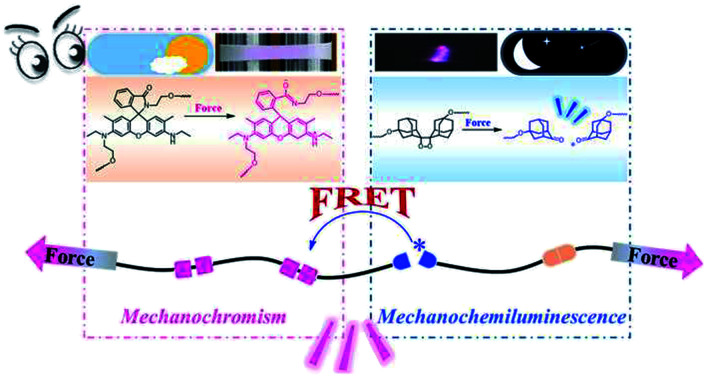

## Introduction

Stress induced covalent bond scission underlies the macroscopic failure of polymeric materials.^1^ The possibility to monitor mechanical stress and deformation at broader time and force scales is of fundamental research interest.^2–5^ The past few decades have witnessed great progress on polymer mechanochemistry, which opens a new avenue for the design of self-reporting polymers with functional mechanophores as stress probes.^6–9^ Along the polymer backbones or within the crosslinks, these motifs that contain mechanically labile bonds, undergo disruption upon mechanical activation, thereby converting mechanical energy into chemical energy.^10–14^ Particularly, mechanochromic or mechanoluminescent polymers, that can generate visualized signals of absorption or fluorescence under applied force, promise the quantitative and locally resolved detection of stress.^15,16^ Great efforts have been devoted to devising new mechanophores with attractive optomechanical properties upon exerting different types and magnitudes of forces.^17–25^

**Fig. 1:**
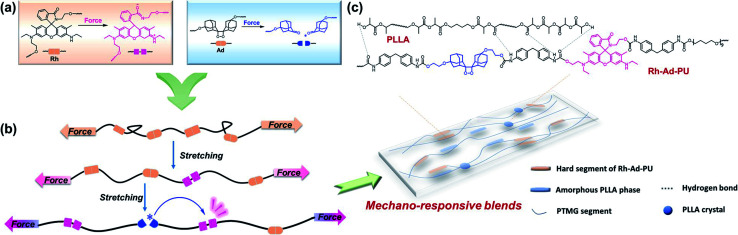
(a) Chemical structures of bis(adamantyl)-1,2-dioxetane (Ad), rhodamine (Rh) and their force activated states. (b) Schematic of mechanically induced breakage of Rh and Ad in a multiblock polymer chain, resulting in mechanochromic and FRET promoted mechanochemiluminescent responses. (c) Chemical structures of PLLA and Rh-Ad-PU; several types of hydrogen-bond interactions in blends are illustrated.

Rhodamine (Rh) and bis(adamantyl)-1,2-dioxetane (Ad) have been demonstrated as versatile self-reporting mechanophores (Fig. 1a).^17,19^ When covalently coupled with polyurethane chains or networks, Rh experiences ring-opening transition under stress.^19,26^ Discoloration and fluorescence thus arise for high-contrast stress detection, since the ring opened Rh exhibits excellent optical properties including a high absorption coefficient and quantum efficiency with long-wavelength absorption and emission. However, exhibiting a more or less persistent mechanochromic response, the non-scissile, reversible Rh delivers limited temporal resolution in recording chain scission events.^26,27^ In this sense, real-time monitoring of bond breakage is possible when bis(adamantyl)-1,2-dioxetane is integrated as a chemiluminescent stress probe.^17^ Mechanical dissociation of the dioxetane ring leads to the excited adamantanone that relaxes to the ground state with blue auto-luminescence (*ca.* 420 nm). Unlike Rh, benefiting from the wide dynamic range of chemiluminescence without external excited sources required, Ad-derivatized polymers feature high spatial and temporal resolution of mechanochemiluminescence, which results in highly localized emission in front of a propagating crack tip.^28^ But due to the low quantum efficiency of the transient blue emission from broken Ad, its mechanical monitoring has to be performed in the dark. Another limitation lies in the high stability of Ad, so that its force threshold is fairly high.^29^

Our motivation was to create more sophisticated mechano-responsive polymers to self-report excessive stress with clearly perceptible signals, meanwhile with the rupture force tailored over a broad range. Rather than synthetic elaborations of mechanophores, combining mechanochromic and mechanochemiluminescent moieties is a straightforward and useful approach.^2^ Firstly, orthogonal optical properties (absorption and luminescence) can be employed for multimodal analysis of damage and stress. Secondly, the force threshold that triggers discoloration and luminescence or the sensitivity of the rate to the applied force can be adjusted facilely.

Considering the complementary advantages of Rh and Ad, together with the large difference in their force free potential energies,^26,29^ we herein incorporated the two distinct mechanophores into polymer blends of polyurethane and polylactic acid that displayed successive occurrences of mechanochromism and mechanochemiluminescence. The rupture of the Rh ring was observed at a low strain, followed by the breakage of Ad, concomitant with intense red emission through effective fluorescence resonance energy transfer (FRET) from excited adamantone to the opened zwitterion of Rh (Fig. 1b). Notably, although the two mechanophores have been well studied individually, they as well as other chromic mechanophores can only respond under specific loading force either under daylight or in darkness.^30–36^ Using this strategy, limitations derived from the large force threshold of Ad and limited resolution of Rh have been overcome, empowering the polymer blends with abilities to report whether, where and when mechanical events take place, which can work as a full-day stress sensor responsive in an expanded force region.

## Results and discussion

As shown in Fig. 1c, the polymer blends are composed of dual mechano-responsive polyurethane (Rh-Ad-PU) as a matrix and hydroxyl terminated low molecular weight polylactic acid (PLLA) as a polymeric filler. With the availability of bis-hydroxyl functionalized Rh and Ad, Rh-Ad-PU containing the two mechanophores (molar ratio = 1 : 1) in the main chain was synthesized through mild polycondensation (synthetic details are described in ESI, Fig. S1, S2 and S5†). Upon fracture, the Rh-Ad-PU film changed its color from colorless to red slightly (Fig. S6a†). Meanwhile, if tested in the dark, the film emitted weak red light (Fig. S6b†). Such optical alterations from Rh-Ad-PU facilitate its utility as self-reporting stress probe in a complementary manner, that is valid both under daylight and in the dark. The Rh-Ad-PU with dual-responsiveness was further explored by integrating it into polymer blends whose mechanical properties and rupture force can be tuned over a broad range. Through this method, the self-reporting performance of the blends is expected to be highlighted, since their broadly tunable mechanical properties would facilitate the mechano-activation of the two mechanophores at distinctly different thresholds.

For that, PLLA existing in the forms of crystalline and amorphous phase in blends was selected as a polymeric filler. The chemical and physical features of PLLA play an important role in the ultimate performance profile of the multiphase blends: (1) the crystalline domains of PLLA with high modulus and their orientation behaviors under applied force can reinforce the blends with increased tensile strength and toughness;^37–39^ (2) the amorphous parts facilitate the formation of hydrogen bonds with polyurethane and the ordering of the amorphous phase is responsive and tunable under tension, which are effective in toughening polyurethane.^37,40^ Translucent polymer blends PU/PLLAs with varied ratios of PLLA from 0 to 20 wt% were prepared *via* the solution casting method (details of the preparation are described in ESI, Table S1†). The transparency of the blends decreased with the increase of the crystalline PLLA content (Fig. S7†), indicating an increase of phase separation between PU and PLLA. This result agreed with the morphological observations by transmission electron microscopy. The phase separation of PU itself was not very obvious, with a phase size of only tens of nanometers (Fig. S8a†), which could be ascribed to the weak aggregation of hard segments,^41^ whereas all the blends displayed a distinct two-phase morphology with the crystalline phase domains which became significant from PU/PLLA-5% to PU/PLLA-20% (Fig. S8b–d†). Besides, DSC measurements supported that all the blends were elastomers, as evidenced by their low glass transition temperature (*T*_g1_ < 0 °C, Fig. S9†). In detail, *T*_g_ associated with the Rh-Ad-PU-rich phase first increased (from −26 °C for PU to −15 °C for PU/PLLA-5%) mainly due to the restricted chain movement by hydrogen bonding between Rh-Ad-PU and PLLA; then, shifted to a lower temperature (−18 °C for PU/PLLA-10%, −20 °C for PU/PLLA-20%) with the emergence of the melting peak for PLLA at higher PLLA content, which could be ascribed to the increase of immiscibility between polyurethane and PLLA crystals.^42–44^

**Fig. 2:**
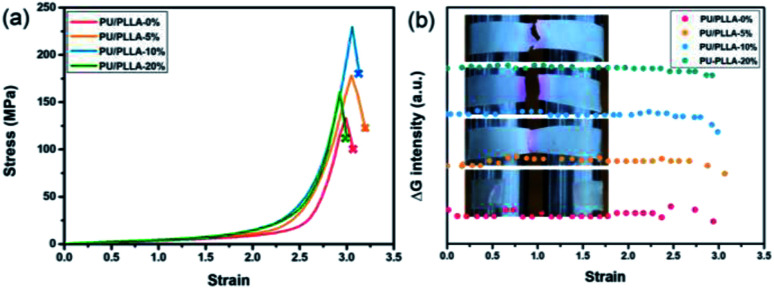
(a) Stress–strain curves and (b) the changes in the green channel upon straining different films at a strain rate of 1.0 s^−1^; insets show the fracture of polymer films.

The polymer films were then subjected to optomechanical studies on a rheometer equipped with an Expansion Instruments SER Universal Testing Platform, and in connection with a camera to record the discoloration and luminescence information (details in the ESI†). As shown in Fig. 2a, the rupture stress of these blends increased prominently compared with PU/PLLA-0%, which was considered to be associated with the reinforced effect of PLLA. Among them, PU/PLLA-10% with medial size of phase domain exhibited superior mechanical properties. As for PU/PLLA-20%, further increasing the PLLA loading imposed a negative effect on its mechanical response. This was probably owing to the increased PLLA crystalline domains that led to the decrease of compatibility of PLLA and polyurethane. Such changes in mechanical properties reflected the importance of the balance between phase separation and crystallinity in these mutliphase polymer systems.^44–46^ Meanwhile, PU/PLLAs are mechanochromic, as can be readily distinguished by the naked eye (Fig. 2b). The ruptured, colored films displayed a characteristic absorption peak (550 nm) of the planar zwitterion from Rh; meanwhile, a fluorescence band peaking at 590 nm was detected when the broken film was excited with a 420 nm light source, corresponding to the ring-opening of Rh under mechanical force (Fig. S10†).^19^ Moreover, since the mechanochromic performance was stress-sensitive, samples with higher rupture stress showed more pronounced discoloration. This finding is essential for Rh as a stress sensor in the blends. A more quantitative study of chromism was done by calculating the RGB ratios of the sequential images extracted from the videos (Fig. S11†). The bandpass of the green channel is well aligned with the absorption band of the zwitterion isomer of Rh.^47^ Herein, the green channel was used to analyze the extent of discoloration. As for the blends PU/PLLAs, the change of the green channel intensity increased along with their tensile stress (Fig. 2b). However, the failure of control specimen Control-PU/PLLA-10% (Table S1†) did not present appreciable changes (Fig. S12†). Such comparison also proved that the green channel was related to the generation of red color in a mechano-chemical manner.

**Fig. 3:**
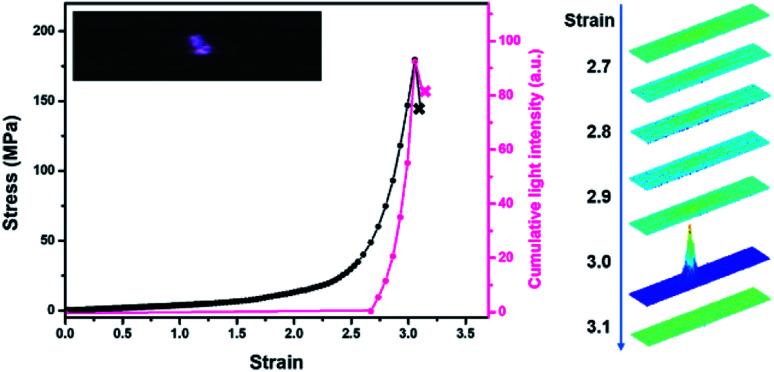
Stress and cumulative light intensity *vs.* strain during stretching of the PU/PLLA-10% film at a strain rate of 1.0 s^−1^. The right graph corresponds to intensity analysis of the stretched sample. The analysed intensity is based on the same region within the sample.

In darkness, transient intense light from the stretched blends was recorded by using a high-speed camera. The evolution of mechanoluminescence from a representative film demonstrated its high spatial and temporal resolution (Fig. 3). Besides, the light intensity in the 5 ms time span of the fracture frame relative to total light intensity is about 41%, manifesting a high concentration of molecular disruption occuring at the fracture site. In relative to polyurethane containing 1,2-dioxetane as the only mechanophore, the emission color from PU/PLLAs varied from blue to red with a remarkable increase in light recognition.^48^ This unique mechanoluminescence is attributed to the large difference in stability and mechano-responsive behaviors between the two distinct mechanophores (discussed below).

**Fig. 4:**
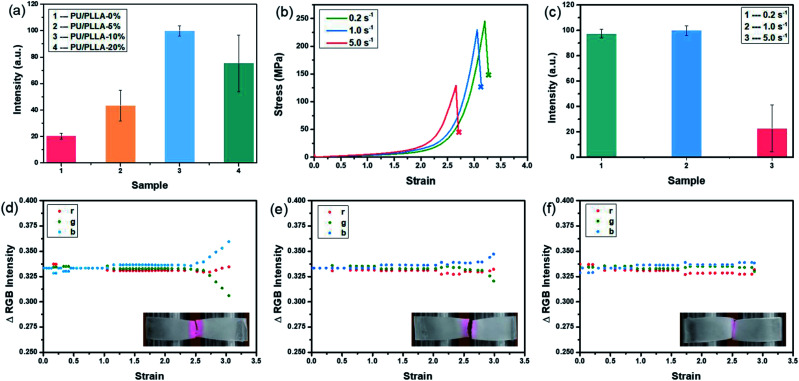
(a) Total light intensity emitted upon stretching PU/PLLA films at a strain rate of 1.0 s^−1^. (b) Stress–strain curves; (c) total light intensity emitted upon stretching films of PU/PLLA-10% at different strain rates. Relative change in RGB intensity upon stretching films of PU/PLLA-10% at a strain rate of (d) 0.2 s^−1^; (e) 1.0 s^−1^; (f) 5.0 s^−1^; insets show the fracture of PU/PLLA-10% films.

To gain a better understanding of the mechanochemical transduction from these self-reporting blends, the effects of PLLA content and strain rate on the mechanochromism and mechanoluminescence were studied. The total light intensity was found to correlate with mechanical stress. As shown in Fig. 4a, the cumulative intensity from PU/PLLA-10% was about 5 times higher than that of PU/PLLA-0% (Rh-Ad-PU without PLLA), despite the diluting effect of inactive PLLA in the blends. Compared to the mechanochromic variation (Fig. 2b), the effect of the PLLA content on the increment of luminescence was increased, which mainly benefited from the FRET procedure from broken Ad to the zwitterion of Rh. The boosted intensity strengthens the self-reporting ability of the blends with improved resolution and sensitivity.

Following this, taking PU/PLLA-10% for example, the strain-rate-dependent mechanical response was estimated. Fig. 4b shows that decreasing the strain rate from 5.0 s^−1^ to 0.2 s^−1^ increased the mechanical properties dramatically in terms of strength and toughness. Also, the slower strain rate on the sample led to a more significant color change and luminescence intensity (Fig. 4c–f, Video S1 and S2†). Since the intensity of red light was proportional to the number of activated mechanophores, these results supported that, at a lower strain rate, mechanochemical transduction was more efficient, so that more Rh and Ad were activated in our blends. Generally, for most non-microphase-separated polymers, chain disentanglement is slowed down effectively at high tension rates, leading to more efficient mechanochemical transition.^49^ In our study, such discrepancy with respect to the negative strain-rate related mechanical responsive behaviors might arise from other unique interactions. The disassociation of numerous hydrogen bonds can remarkably consume the strain energy, and contributes to the significant improvement of toughness.^50^ Besides, strain-induced orientation is a well-known characteristic of PLLA reinforced elastomers.^37,38,40^ The two factors are kinetically controllable, which is favored under low strain rate, but negligible for ameliorating mechanical properties as well as for bond scission events at high rates.^51^ As shown in Fig. S13,† after slow stretching, the film orientation lying parallel to the tensile axis was observed.

Notably, although Ad in the force free state seems much more stable than Rh (*E*_a_: 154.9 kJ mol^−1^*vs.* 96.2 kJ mol^−1^),^26,29^ their chemical conversion under tension is unpredictable. Revealing the competitive mechanochemical process of Ad and Rh is difficult in theory. This is because the mechanochemiluminescence of dioxetane follows the non-adiabatic dissociation mechanism that largely precludes the well-used quantum-chemical calculations of force-dependent kinetics.^27^ Pleasingly, herein experimentally, we found the successful FRET process under tension (Fig. S10†), which in turn indicated a very likely, successive bond scission process from the two mechanophores. That is, Rh units are more labile; part of them are mechanically activated ahead of the breakage of Ad, and serve as the fluorescent acceptor of the excited ketone to emit bright red light.

**Fig. 5:**
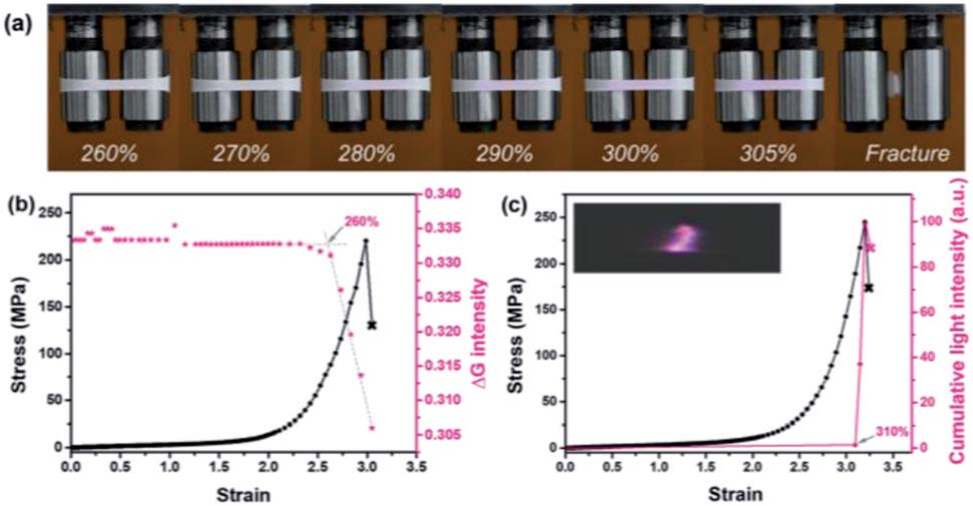
(a) Pictures illustrating mechanochromism under deformation. (b) Stress and the intensity in the green channel as a function of strain. (c) Stress and cumulative light intensity as a function of strain according to the analysis of representative mechanochromic and mechanoluminescent videos (the PU/PLLA-10% film was stretched at a strain rate of 0.2 s^−1^).

When different mechanophores were coupled closely, a reduction in their mechano-activation was very likely anticipated since they shared the overall stress at separate sites.^10,13,52^ To elaborate on this point, reference polymer blends containing Rh or Ad only (Rh-PU/PLLA-10% and Ad-PU/PLLA-10%) were prepared for optomechanical studies (Table S1, Fig. S1, S3 and S4†). As shown in Fig. S14,† the discoloration of Rh-PU/PLLA-10% and luminescence of Ad-PU/PLLA-10% set on at the deformation of *ca.* 278% (*ca.* 79 MPa) and *ca.* 357% (*ca.* 214 MPa), respectively. On the other hand, Fig. 5b and c shows the functions of chromism and light intensity *vs.* strain for PU/PLLA-10% (Video S1 and S2†). As for the bis-mechanophore counterpart, the chromism appeared at a strain of *ca.* 260% (*ca.* 80 MPa); further elongation until a strain of *ca.* 310% (*ca.* 180 MPa) triggered the activation of Ad. Valuable information deduced from these data is as follows: (1) a distinct difference in the mechanical activation between Rh-PU/PLLA-10% and Ad-PU/PLLA-10% was indeed observed, which supported that many of the Rh units could be mechanically activated before Ad. (2) The existence of an overlapping stress region for chromism and luminescence implicated the simultaneous breakage of a fraction of the two mechanophores after many of the Rh rings were opened under low stress. (3) The force thresholds for the respective activation of Rh-PU/PLLA-10% and Ad-PU/PLLA-10% were similar to those of PU/PLLA-10%, except for the slight “shifting” of the chemiluminescent transition to the lower strain in our bis-mechanophore system mainly due to the additional FRET process to sensitize the luminescence signal. Therefore, our results indicated that the difference between Rh and Ad didn't translate into an observable impact on the activation of PU/PLLA-10% in the bulk state, or the mechanochemical coupling effect on the scission rate of adjacent Rh and Ad was not sufficient enough to be detected.^52^

Additionally, through this coupling method, not only a versatile analysis of material damage can be realized *via* two different manifestations, but also the stress history can be captured, in that the distinctive change of chemiluminescence color from blue to red recorded a prior force-loading on the Rh moieties (Fig. 5a). Importantly, since the two specific bond rupture events can be stimulated at highly different mechanical levels, expanding the responsive force range from these mechano-sensing blends now can be realized. Overall, the limitation arising from the high stability of the mechanochemiluminescent polymers with Ad alone now can be overcome, with effectively “shifting” the appreciable optical changes to a much lower force threshold, and meanwhile exhibiting a more dynamic change upon the deformation process. This finding is highly helpful for probing the stress evolution of the damaged samples over a broader force range with high resolution that was not able to be addressed previously.

## Conclusion

We have developed a new class of stress-reporting PU/PLLA blends by merging dual distinct mechanochromic and mechanochemiluminescent units (Rh and Ad) into polymer main chains. The two mechanophores can be activated and exhibit red discoloration and red chemiluminescence, respectively, according to the different strengths of mechanical stimuli. Taking advantage of the two mechanophores, the yielded macroscopically detectable optical signals from these blends have been proved as stress-dependent, with high resolution to report the evolution and localization of damage at the molecular scale, which cannot be realized by using one mechanophore alone. Furthermore, the fact that mechanically generated discoloration takes place obviously earlier than that of luminescence, not only indicates an expanded responsive force range, but also in turn, confirms that the activation energy of Rh is less than that of Ad under mechanical conditions. Our strategy thus is applicable for the comparison of the bond energy of different mechanophores, even if it is difficult theoretically. Also, setting aside the enormous synthetic efforts on new multi-functional mechanophores, the present approach is versatile in unravelling and enhancing mechanochemical processes at the molecular level; thus, it can facilitate the full-day stress detection in a mutimodal analysis.

## Conflicts of interest

There are no conflicts to declare.

## Supplementary Material

SC-012-D0SC06140A-s001

SC-012-D0SC06140A-s002

SC-012-D0SC06140A-s003
