# Supplementary material for: Empowering self-reporting polymer blends with orthogonal optical properties responsive in a broader force range
Source: Chem Sci. 2020 Dec 8;12(4):1245–50. doi: 10.1039/d0sc06140a (PMC8179123; doi:10.1039/d0sc06140a)
Supplement: SC-012-D0SC06140A-s001 [file SC-012-D0SC06140A-s001.pdf]

Electronic Supplementary Material (ESI) for Chemical Science.
This journal is © The Royal Society of Chemistry 2020

Supporting Information for

Empowering Self-Reporting Polymer Blends with Orthogonal Optical Properties Responsive at Broader Force Range

Mengjiao Wu,[a] Zhen Guo,[a] Weiye He,[a] Wei Yuan[a] and Yulan Chen*,[a]

[a] Department of Chemistry, Key Laboratory of Mechanism Theory and Equipment Design of State Ministry of Education, Tianjin University, Tianjin, 300354, China

*E-mail: yulan.chen@tju.edu.cn

Phone/Fax: +86-22-27404118

Table of Contents

1. General Experimental Details

Materials……………………………………………………………………S3
Characterization methods……………………………………………………S3
Testing of mechanochromism…………………………………………………S4
Optomechanical testing………………………………………………………S4
RGB color analysis……………………………………………………………S4

2. Synthetic Procedures for PU/PLLA Blends

Synthesis of dual mechano-responsive linear polyurethane (**Rh-Ad-PU**)………..S5
Synthesis of mechano-responsive linear polyurethanes (**Rh-PU**, **Ad-PU**)……………………………………………………………………...S5
Synthesis of control linear polyurethane (**L-Blank-PU**)……………………………..S6
Synthesis of low molecular weight polylactic acid (**PLLA**)………………………....S7
General preparation method of **PU/PLLA** blend polymer films……………………..S7

3. Supplementary Table…………………………………………………………...S8

4. Supplementary Figures

Figure S1. The GPC curves of linear polyurethanes………………………………….S8
Figure S2. ^{1}H NMR spectrum of **Rh-Ad-PU** in $CDCl_3$………………………………S9
Figure S3. ^{1}H NMR spectrum of **Rh -PU** in $CDCl_3$…………………………………S9
Figure S4. ^{1}H NMR spectrum of **Ad-PU** in $CDCl_3$………………………………S10
Figure S5. ^{1}H NMR spectrum of **PLLA** in $CDCl_3$………………………………S10
Figure S6. The images of fractured **Rh-Ad-PU**……………………………………S11
Figure S7. UV-vis transmittance of **PU/PLLA** blends……………………………S11
Figure S8. TEM images of **PU/PLLA** blends…………………………………..S11
Figure S9. DSC curves of **PLLA**, **Rh-Ad-PU** and blends…………………………S12
Figure S10. UV-vis absorption spectrum and FL spectra of **PU/PLLA-10%**………S12
Figure S11. RGB analysis for **PU/PLLA** blends…………………………………S13
Figure S12. RGB analysis for **Control-PU/PLLA-10%** at different strain rates…...S13
Figure S13. SEM images of **PU/PLLA-10%** surface before and after stretching…..S14
Figure S14. Mechanochromic and mechanoluminescent analysis of **Rh-PU/PLLA-10%** and **Ad-PU/PLLA-10%**……………………………………………………S14
Figure S15. Setups for mechanochromism and mechanoluminescence tests……....S15

5. References…………………………………………………………………S15

1. General Experimental Details

Materials. Unless otherwise stated, all regents were purchased from commercial sources and used without further purification. All reactions were performed under argon atmosphere unless otherwise specified, and all glass wares were oven dried before use. 5,5′/7′-(2-Hydroxyethylenoxy)adamantylideneadamantane 1,2-dioxetane (**Ad**), 3'-(ethyl(2-hydroxyethyl)amino)-6'-(ethylamino)-2-(2-hydroxyethyl)-2',7'-dimethylspiro-[isoindoline-1,9'-xanthen]-3-one (**Rh**) were prepared according to the previous literatures.[S1,S2] Polytetramethylene glycol (**PTMG**: Mn = 650 g/mol) was dried at 70 ºC under vacuum for 2 hours before use. Chloroform ($CHCl_3$) was distilled under argon over CaH_2 prior to use.

Characterization methods. Liquid ^1H NMR spectra were recorded in $CDCl_3$ solvent on a Bruker AVANCE III-400 spectrometer. Differential scanning calorimetry (DSC) measurements were conducted using the TA Instruments Q-20 with a scan rate of 10 °C/min under nitrogen atmosphere. Field emission scanning electron microscopies (FE-SEM) were carried out using a Hitachi Limited model SU800 microscope operating at an accelerating voltage of 3.0 kV. Gel Permeation Chromatography (GPC) of the PU samples was carried out an Agilent PL-GPC50 with multi detectors, using tetrahydrofuran (THF) as an eluent with a flow rate of 1.0 mL/min. The molecular weights of the tested polymers were determined by the light scattering detector. UV-vis absorption spectra were obtained on a PerkinElmer Lamda 750 spectrophotometer. Fluorescence spectra were recorded on a Hitachi F-7000 fluorescence spectrophotometer. High resolution transmission electron

microscopies (HR-TEM) were performed on a JEOL model JEM-2100 plus microscope operating at an accelerating voltage of 200 kV. The ultrathin specimens were prepared by LEICA EM UC7.

Testing of mechanochromism. Tensile experiments were carried out on a TA Rheometrics, DHR-2 equipped with an Xpansion Instruments, SER3, extensional fixture. Optical images were captured by Nikon D5500 with 18-55mmf/3.5-5.6G VR lens under ambient room light (Figure S15a). The pictures were taken every 20 ms from the starting point of each tensile test. The dimension of the strips used for tensile tests was 30 × 5.3 × (0.20 ± 0.03) mm.

Optomechanical testing. Tensile experiments were carried out on a TA Rheometrics, DHR-2 equipped with an Xpansion Instruments, SER3, extensional fixture. The two rotating drums of the fixture are colored black by permanent marker to eliminate reflecting light. The pco.edge 5.5 camera equipped with a Nikon AF NIKKOR 50 mm 1:1.4D lens was used to record videos in darkness (Figure S15b). All the videos were recorded in the rolling shutter color mode with a shooting rate of 200 fps and exposure time of 5.00 ms. The frames of the resulting video were exported as separate monochrome TIF-files and light intensity was analyzed with a homemade program in MATLAB as literature.[S3] The total intensity for a dark image as the noisy signal was subtracted from all film intensities. The dimension of the strips used for tensile tests was 30 × 5.3 × (0.20 ± 0.03) mm.

RGB color analysis

The RGB value was obtained by analyzing images in MATLAB software. RGB ratios

of each image was calculated using Grassmann's law.

$$r = \frac{R}{R+G+B};\ g = \frac{G}{R+G+B};\ b = \frac{B}{R+G+B}$$

r, *g*, and *b* are the red, green and blue component of the RGB ratio, respectively. R, G and B are the average intensity of red, green and blue channels in the region of interest.

2. Synthetic Procedures for PU/PLLA Blends

Synthesis of dual mechano-responsive linear polyurethane (Rh-Ad-PU).

The dibutyltin dilaurate (17 μL) in 2 mL $CHCl_3$ was added to a mixture of diphenyl-methane-diisocyanate (MDI, 1.45g, 5.79 mmol), 5,5'/7'-di(2-hydroxyethylenoxy) adamantylideneadamantane 1,2-dioxetane (**Ad**, 109.82 mg, 0.26 mmol), 3'-(ethyl(2-hydroxyethyl)amino)-6'-(ethylamino)-2-(2-hydroxyethyl)-2',7'-dimethylspiro[isoindoline-1,9'-xanthen]-3-one (**Rh**, 131.0 mg, 0.26 mmol) and $CHCl_3$ (10 mL), the solution was stirred 30 min at 30°C under an argon atmosphere. Then the polytetramethylene glycol (**PTMG**, M_n = 650 g/mol, 3.4g, 5.23 mmol) in $CHCl_3$ (10 mL) was added. After stirring for another 1.5 hours at 45 °C, the reaction mixture was cooled down to room temperature and precipitated into *n*-hexane. The precipitate was collected and dried in vacuo at room temperature to afford **Rh-Ad-PU** as off-white rubbery solid (*M*n = 62.0 kDa, *M*w = 207.5 kDa, PDI = 3.35, Figure S1). The chemical compositions of the **Rh-Ad-PU** were determined by ^{1}H NMR (Figure S2). **Rh** and **Ad** were successfully coupled into polyurethane chains in a covalent way, as confirmed by the appearance of characteristic peaks at 8.00 and 2.82 ppm, respectively.

Synthesis of dual mechano-responsive linear polyurethanes (Rh-PU or Ad-PU).

The dibutyltin dilaurate (9 μL) in 1 mL $CHCl_3$ was added to a mixture of diphenyl-methane-diisocyanate (MDI, 0.69g, 2.77 mmol), 5,5'/7'-di(2-hydroxyethylenoxy) adamantylideneadamantane 1,2-dioxetane (**Ad**, 54.91 mg, 0.13 mmol) or 3'-(ethyl(2-hydroxyethyl)amino)-6'-(ethylamino)-2-(2-hydroxyethyl)-2',7'-dimethylspiro[isoindoline-1,9'-xanthen]-3-one (**Rh**, 65.5 mg, 0.13 mmol) and $CHCl_3$ (5 mL), the solution was stirred 30 min at 30 °C under an argon atmosphere. Then the polytetramethylene glycol (**PTMG**, M_n = 650 g/mol, 1.7 g, 2.62 mmol) in $CHCl_3$ (5 mL) was added. After stirring for another 1.5 hours at 45 °C, the reaction mixture was cooled down to room temperature and precipitated into *n*-hexane. The precipitate was collected and dried in vacuo at room temperature to afford **Rh-PU** or **Ad-PU** as off-white rubbery solid (**Rh-PU**, *M*n = 83.1 kDa, *M*w = 328.1 kDa, PDI = 3.95; **Ad-PU**, *M*n = 72.3 kDa, *M*w = 172.2 kDa, PDI= 2.38, Figure S1). The chemical compositions of the **Rh-PU** or **Ad-PU** were determined by ^{1}H NMR (Figure S3-S4). **Rh** or **Ad** were successfully coupled into polyurethane chains in a covalent way, as confirmed by the appearance of characteristic peaks at 8.00 or 2.82 ppm, respectively.

Synthesis of control linear polyurethane (L-Blank-PU).

The dibutyltin dilaurate (17 μL) in 2 mL $CHCl_3$ was added to a mixture of diphenyl-methane-diisocyanate (MDI, 1.45g, 5.79 mmol), and $CHCl_3$ (10 mL). Then the polytetramethylene glycol (PTMG, M_n = 650 g/mol, 3.74g, 5.75 mmol) in $CHCl_3$ (10 mL) was added. After stirring for another 1.5 hours at 45 °C, the reaction mixture was cooled down to room temperature and precipitated into *n*-hexane. The precipitate was collected

and dried in vacuo at room temperature to afford **L-Blank-PU** as off-white rubbery solid (Mn = 142.4 kDa, Mw = 333.96 kDa, PDI = 2.35, Figure S1).

Synthesis of low molecular weight polylactic acid (PLLA).

PLLA was prepared via one-step ring-open polymerization. L-lactide (1.66 g, 11.5 mmol) and 1,4-butanediol (45.06 mg, 0.5 mmol) were added into a two-necked flask under argon atmosphere. After heating to 125 °C, the mixture was melted. Then, $Sn(Oct)_2$ (13.98 mg, 0.035 mmol) with tetrafluoride tablets was added and the reaction was kept at 160 °C for 2 hours. The reaction mixture was cooled down to room temperature, followed by adding dichloromethane. The mixture was then deposited in cold methanol. The precipitate was collected and dried in vacuo at room temperature to afford **PLLA** as a white solid powder (1.0 g). The molecular weight of **PLLA** (M_n = 4.3 kDa) was characterized by ^{1}H-NMR as shown in Figure S5 and calculated through formula.[S4]

$$Mn = \left(\frac{I_1}{I_2} + 1\right) * 72 * 2 + 90 \quad (1)$$

Where I_1 and I_2 are the areas of peaks 1 and 2, respectively, 72 is the molar mass of one lactic acid repeat unit, and 90 is the total molar mass of the rest part of the molecule.

General preparation method of PU/PLLA blend polymer films.

The as-prepared **Rh-Ad-PU**, **Rh-PU**, **Ad-PU** or **L-Blank-PU** respectively, was added to tetrahydrofuran (THF) and stirred until a uniform solution was obtained. Meanwhile, different amount of **PLLA** (Table S1) were added into THF respectively and stirred until the powders were fully dissolved. Then, the two polymer solutions were mixed together and stirred for 3 hours before poured into a Teflon mould (50 × 30 × 10 mm). THF was

evaporated under ambient conditions followed by vacuum drying of the films at room temperature for 12 hours. The removal of THF was confirmed by DSC analyses (Figure S9).

3. Supplementary Table

Table S1. Feed Ratios of Polymer Films in This Study.

Samples	Rh-Ad-PU (mg)	PLLA diol (mg)	PLLA Contents (wt%)	L-Blank-PU (mg)
PU/PLLA-0%	400	0	0	0
PU/PLLA-5%	400	21	5	0
PU/PLLA-10%	400	45	10	0
PU/PLLA-20%	400	100	20	0
Control-PU/PLLA-10%	0	45	10	400
Rh-PU/PLLA-10%	400	45	10	0
Ad-PU/PLLA-10%	400	45	10	0

4. Supplementary Figures

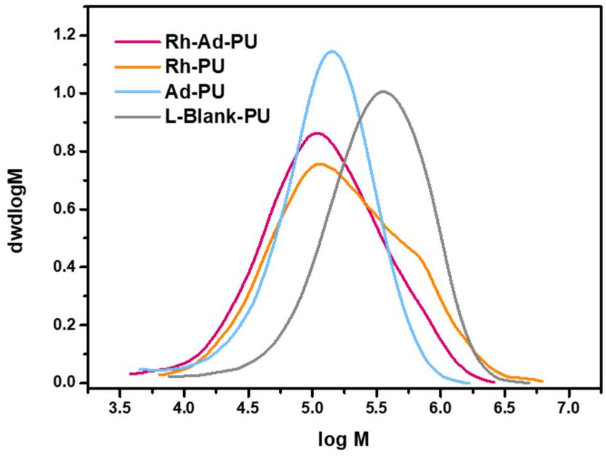

Figure S1. The GPC traces of **Rh-Ad-PU**; **Rh-PU**; **Ad-PU** and **L-Blank-PU**.

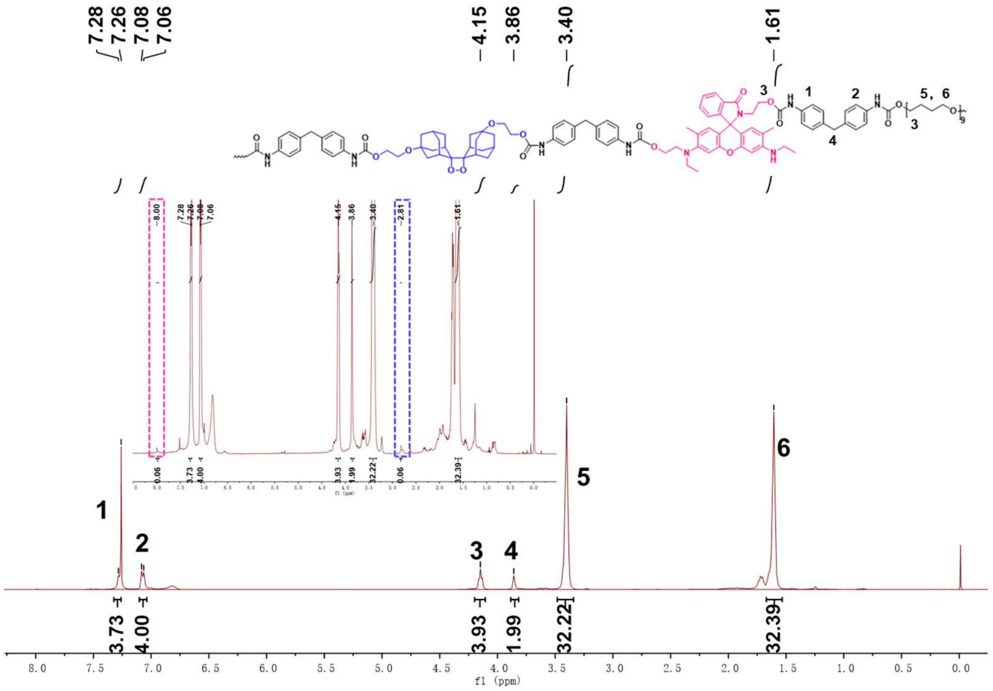

Figure S2. ^{1}H NMR spectrum of the **Rh-Ad-PU** in $CDCl_3$.

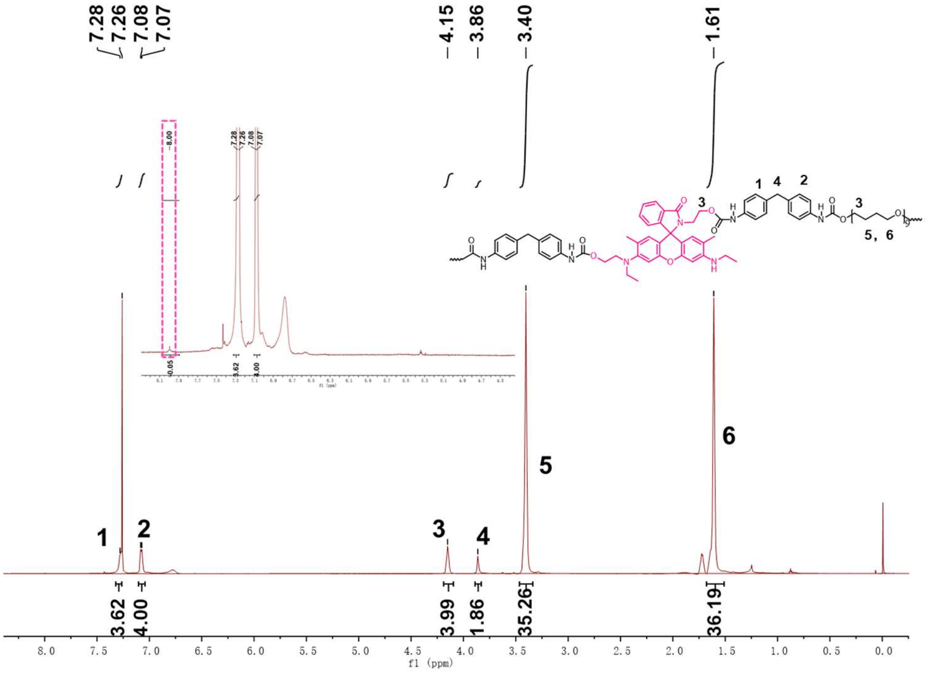

Figure S3. ^{1}H NMR spectrum of the **Rh-PU** in $CDCl_3$.

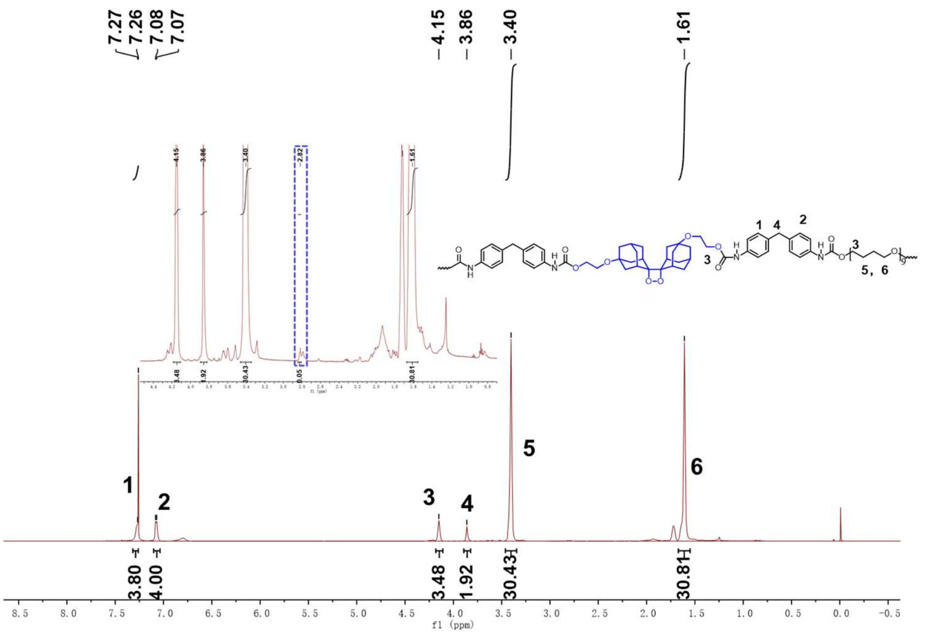

Figure S4. ^{1}H NMR spectrum of the **Ad-PU** in $CDCl_3$.

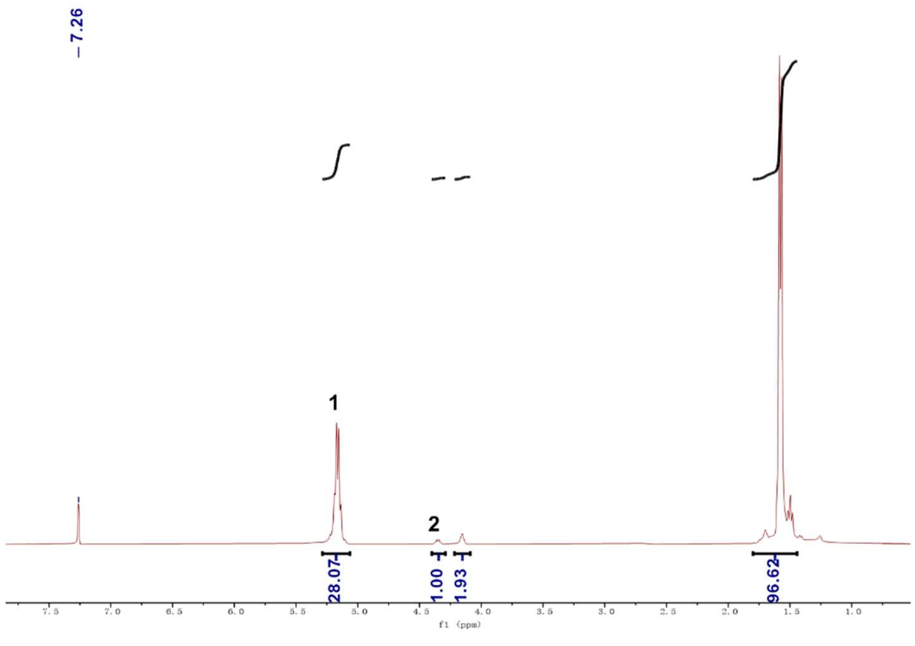

Figure S5. ^{1}H NMR spectrum of the **PLLA** in $CDCl_3$.

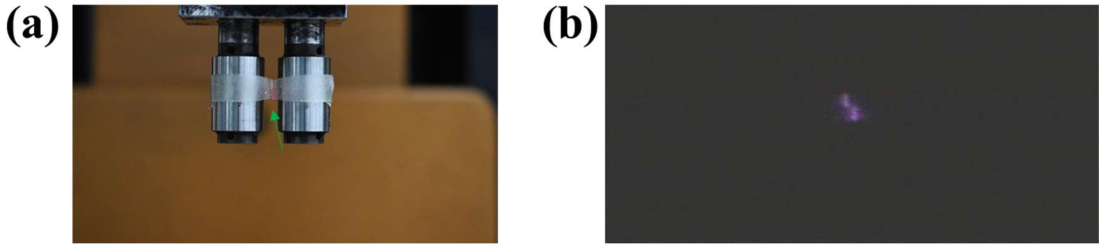

Figure S6. Images of the ruptured **Rh-Ad-PU** film: (a) under daylight and (b) in the dark.

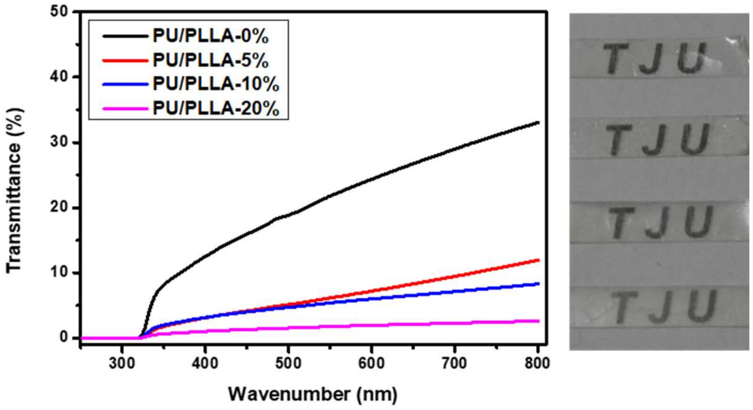

Figure S7. UV-vis transmittance of **Rh-Ad-PU** and its blended films. Insert shows four pieces of films on a labeled paper.

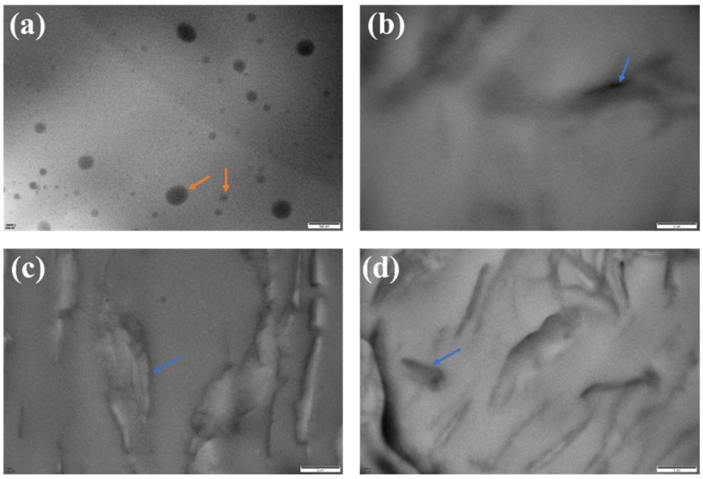

Figure S8. TEM images of ultrathin specimens (thickness: ca. 50 nm) cut at cryogenic conditions from bulk samples of (a) **PU/PLLA-0%** (scale: 500 nm); (b) **PU/PLLA-5%** (scale: 2 μm); (c) **PU/PLLA-10%** (scale: 2 μm) and (d) **PU/PLLA-20%** (scale: 2 μm).

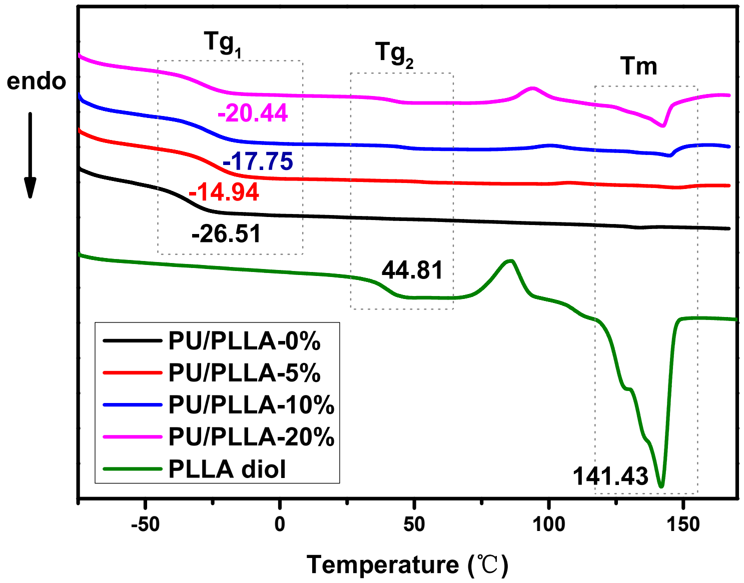

Figure S9. DSC curves of **PLLA**, **Rh-Ad-PU** and its blends (The glass transition temperature is obtained by TA data processing software).

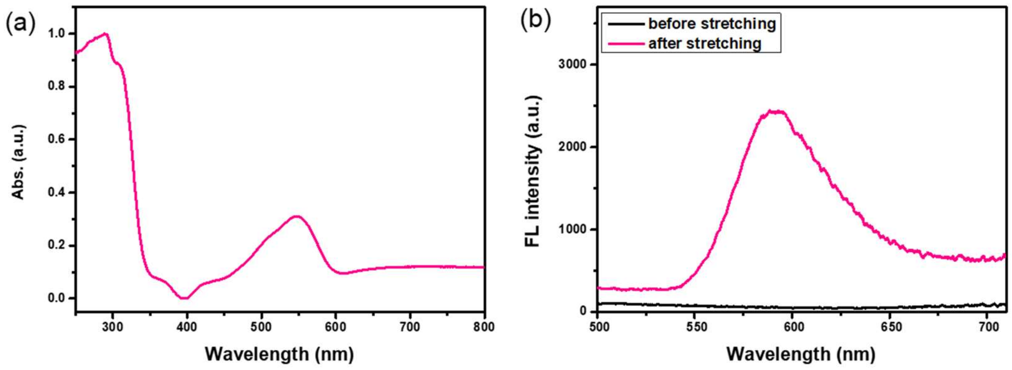

Figure S10. (a) UV-vis absorption spectrum of a **PU/PLLA-10%** film after stretching; (b) FL spectra of a **PU/PLLA-10%** film before and after stretching (Excitation wavelength: 420 nm). The ruptured film exhibited absorption at 420 nm and emitted red fluorescence, due to the existence of ring opened **Rh**,[S2] that showed its potential as the fluorescent acceptor of excited adamantone (λ_{em} = 420 nm).[S5]

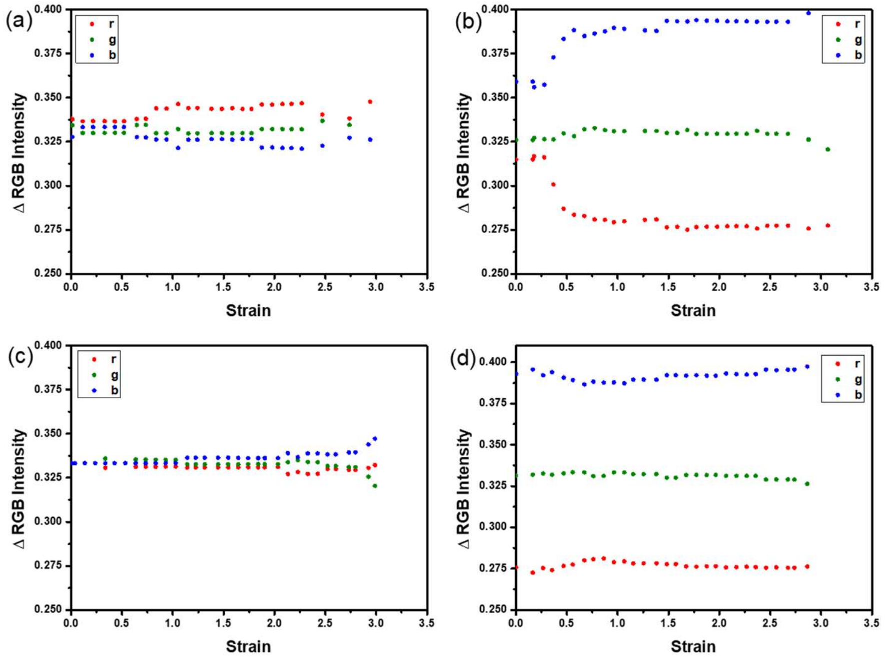

Figure S11. RGB analysis for blends of (a) **PU/PLLA-0%** (b) **PU/PLLA-5%** (c) **PU/PLLA-10%** (d) **PU/PLLA-20%**. All the tests were conducted under daylight lamp.

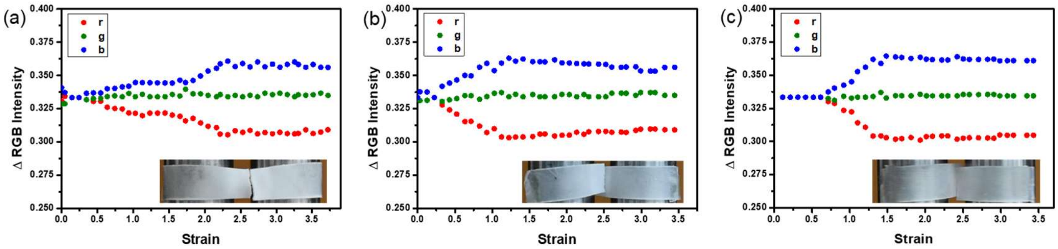

Figure S12. RGB analysis for **Control-PU/PLLA-10%** at (a) 0.2 s^{-1} (b) 1.0 s^{-1} (c) 5.0 s^{-1} strain rate. All the tests were conducted under daylight lamp. The illustrations are the pictures of the fractured blends.

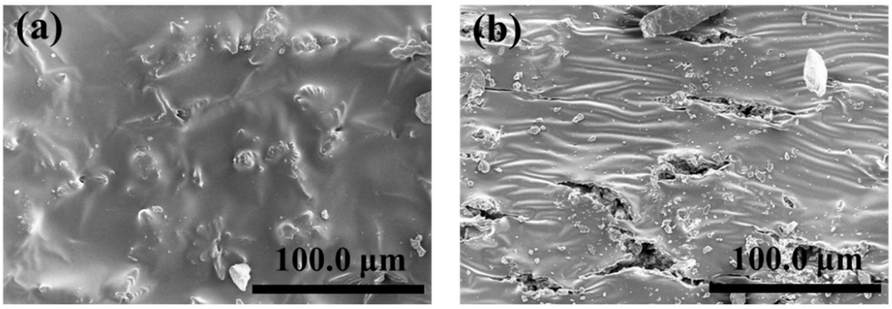

Figure S13. SEM images of **PU/PLLA-10%** surface (a) before and (b) after stretching.

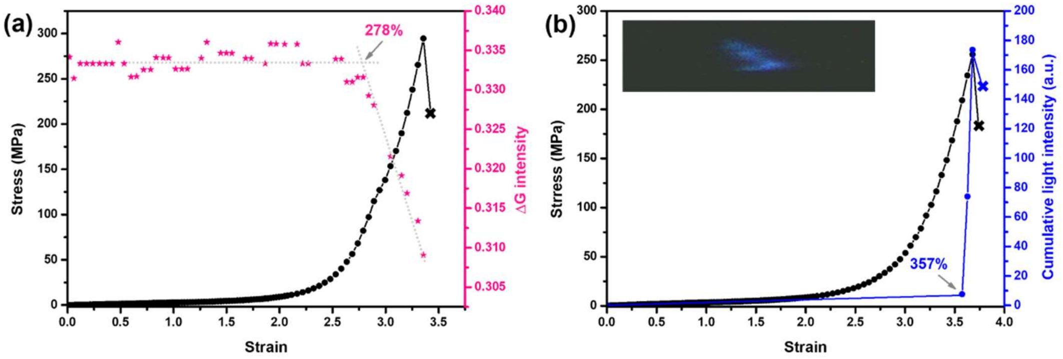

Figure S14. (a) Stress and the intensity in green channel as a function of strain (The **Rh-PU/PLLA-10%** film was stretched at a strain rate of 0.2 s^{-1}). (b) Stress and cumulative light intensity as a function of strain (The **Ad-PU/PLLA-10%** film was stretched at a strain rate of 0.2 s^{-1}).

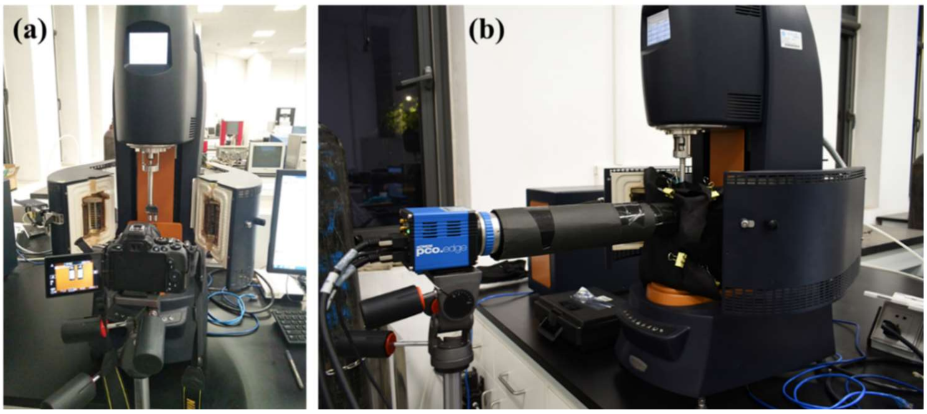

Figure S15. Setups for the mechanochromic and mechanoluminescent tests recorded (a) under daylight; (b) in the dark.

5. <u>References</u>

S1. Chen, Y.; Spiering, A. J. H.; Karthikeyan, S.; Peters, G. W. M.; Meijer, E. W.; Sijbesma, R. P. Mechanically Induced Chemiluminescence from Polymers Incorporating a 1,2-Dioxetane Unit in the Main Chain. *Nat. Chem*. **2012**, *4*, 559-562.

S2. Wang, T.; Zhang, N.; Dai, J.; Li, Z.; Bai, W.; Bai, R. Novel Reversible Mechanochromic Elastomer with High Sensitivity: Bond Scission and Bending-Induced Multicolor Switching. *ACS Appl. Mater. Interfaces* **2017**, *9*, 11874-11881.

S3. Chen, Y.; Sijbesma, R, P. Dioxetanes as Mechanoluminescent Probes in Thermoplastic Elastomers. *Macromolecules* **2014**, *47*, 3797-3805.

S4. Shi, L.; Zhang, R-Y.; Ying, W-B.; Hu, H.; Wang, Y-B.; Guo, Y-Q.; Wang, W-Q.; Tang, Z-B.; Zhu, J. Polyether-polyester and HMDI Based Polyurethanes: Effect of PLLA Content on Structure and Property. *Chinese J. Polym. Sci.* **2019**, *37*, 1152-1161.

S5. Hummelen, J. C.; Luider, T. M.; Wynberg, H. Functionalized Adamantylideneadamantane 1,2-

Dioxetanes: Investigations on Stable and Inherently Chemiluminescent Compounds as a Tool for Clinical Analysis. *Pure & Appl. Chem.* **1987**, *59*, 639-650.
